# An everyday-task-focused, strategy-based educational program for informal dementia carers: a feasibility and pilot study

**DOI:** 10.1038/s41598-026-36887-3

**Published:** 2026-01-20

**Authors:** Carmen Amato, Gemma Burridge, Rhyannah Lesleighter, Georgina Lunt, Susanna Hill, Danielle Ní Chróinín, Karen P. Y. Liu

**Affiliations:** 1 Department of Rehabilitation Sciences, The Hong Kong Polytechnic University, Hong Kong SAR, China; 2 Liverpool Hospital South Western Sydney Local Health District, Liverpool, NSW Australia; 3 School of Health Sciences, Western Sydney University, Liverpool, NSW Australia

**Keywords:** Activities of daily living, Dementia, Informal carers, Educational program, Feasibility, Health care, Medical humanities, Psychology, Psychology

## Abstract

Informal carers play an essential role in assisting people living with dementia with everyday tasks. However, there is a lack of individualized educational programs that are everyday-task-focused, with useful caring strategies as resources, providing a collaborative approach to support carers to assist with declining performance in everyday tasks for people with dementia. This study aimed to pilot test the feasibility, acceptability and effectiveness of such a program to improve carer burden, quality of life and performance in everyday tasks for their care-recipient. Sixteen carers of people living with dementia participated in the seven-week educational program. The results showed the program’s feasibility, with excellent attendance and retention rates, and positive feedback from the carers showed acceptability. Improved personal growth for carers and increased attention to conversation and shopping for care-recipients were key findings. Our preliminary findings support using an everyday-task-focused, strategy-based educational program to improve dementia carer’s well-being and care-recipients’ performance in everyday tasks.

**Supplementary Information:**

The online version contains supplementary material available at 10.1038/s41598-026-36887-3.

## Introduction

Dementia is a progressive neurodegenerative disease that impacts memory, cognition, communication, behavior and mobility, ultimately affecting an individual’s ability to perform everyday tasks, including Activities of Daily Living (ADLs) and Instrumental Activities of Daily Living (IADLs)^1^. ADLs encompass basic self-care tasks such as dressing and toileting, while IADLs involve more complex activities like cooking and shopping, which are essential for independent living^2^.

In Australia, approximately 71% of people diagnosed with dementia reside in the community^3^, with the majority relying on informal carers – unpaid, often untrained family members -–for assistance with daily activities^4^. As dementia progresses and independence declines, carers face increasing responsibilities, which can lead to significant burden and stress. Amato, et al^5^. reported significant correlations between the burden scale and the difficulties experienced in caring for dressing, toileting, and showering (rho = 0.30–0.75) and most IADLs (rho = 0.29–0.47), potentially impeding their ability to assist their care-recipients with everyday tasks. Supporting carers is crucial, not only for their well-being but also to enable people with dementia to remain in their homes and delay institutional care.

Various interventions exist to support informal carers, including education, training, and psychosocial programs^6^. Evidence suggests that education and training are particularly effective in improving carers’ mental health and coping skills^7,8^. However, most programs focus on managing behavioral symptom or psychological support, with limited emphasis on practical strategies for assisting care-recipients with everyday tasks^9^. Previous studies indicate that targeted education can enhance carers’ ability to support ADLs and reduce burden, but these interventions have typically addressed only a narrow range of activities^10,11^.

To address this gap, we developed a collaborative, individualized, everyday-task-focused, strategy-based educational program for informal carers, grounded in the Montessori approach for Dementia and Ageing^12,13^. The program incorporates over 200 practical strategies to help carers engage care-recipients in meaningful everyday tasks, tailored to individual abilities and preferences. The aim is to provide carers with actionable tools to assist their family member with dementia in everyday tasks and to enhance their own well-being and the quality of life.

This study evaluates the feasibility and acceptability of the strategy-based educational program for informal carers, and explores its preliminary impact on carer burden and quality of life, and care-recipients’ engagement in everyday tasks.

## Methods

As a feasibility study, this study adopted a quasi-experimental, single-group, pre- and post-test study design^14^. Ethical approval was attained from the Western Sydney University Human Research Ethics Committee (H11758) and the South-Western Sydney Local Health District Human Research Ethics Committee (H04566). The study was performed in accordance with the relevant guidelines and regulations. Informed written consent was obtained from all participants to use the data collected before data collection.

### Participants

Participant dyads, including a primary informal carer and care-recipient diagnosed with dementia, were recruited through a hospital in South-West Sydney, Australia. Participants were recruited using a convenience sampling method through the hospital’s geriatric, acute, and general medicine wards. The inclusion criteria were: (i) care-recipients with a diagnosis of dementia; (ii) informal dementia carers over the age of 18 years old; (iii) informal dementia carers who self-reported no diagnosis of cognitive impairment; (iv) the informal dementia carer being the primary caregiver for the care-recipient; and (v) care being provided in the community or independent living setting. Participants were excluded if the care-recipients had a previous non-dementia medical condition impacting their independence in performing everyday tasks.

### Everyday-task-focused, strategy-based educational program (Appendix A)

Program development and implementation were underpinned by three theoretical frameworks or models. The Montessori approach emphases the use of an individualized and collaborative approach to engage individuals in meaningful tasks through their interests, skills, and abilities. Secondly, the Theory of Stress and Coping^15^ was used for informal dementia carers to develop skills, strategies and give them resources to prevent stress. Stress can often occur through the interaction of both environmental challenges and difficulties with their care-recipient. The aim was to develop a positive and favorable response to stressors that may arise in the caring^16^. Lastly, the Consumer and Community Engagement Model^17^ was adopted to develop the educational program. The educational program content and strategies were developed collaboratively with informal dementia carers and tailored to be relevant and meaningful to the care-recipients with dementia^17^. As recommended in the position statement regarding principles of care for dementia, teaching informal dementia carers’ problem-solving skills is essential to the success of the education^18^. The program went through a content validation with 14 occupational therapists as expert panel members and was rated as having good content validity (Content Validity Index ranged from 0.93 to 1.00) (Appendix A).

Informal dementia carers participated in a seven-week educational program involving one educational session, three follow-up phone calls and a home visit or a phone call. The two-hour educational session occurred with the informal dementia carer either in person at the hospital, within their home, or by phone. During the session, a booklet including the strategy-based framework, and a list of strategies were provided and explained. They included strategies from four categories; engage, adapt, orientate and sense^19^. The strategies were also grouped by the types of everyday tasks, with additional general strategies and strategies to support with aggression, agitation, and hallucinations. The access to this resource is a critical part of dementia care for informal dementia carers as recommended in the position statement regarding principles of care for dementia^18^. Informal dementia carers were supported to work through case study examples, using the strategy framework and list of strategies. This process was then applied to participating carers’ individual concerns when assisting with everyday tasks, working collaboratively with program instructors to solve problems. The booklet al.so included a program schedule and a journal to record their experiences to be used in the subsequent weeks.

Following the educational session, carers received four follow-up sessions. Three 20-minute follow-up phone calls were conducted during weeks two, four and six of the program. The phone calls aimed to provide additional support for informal dementia carers in using the framework and strategies introduced during the education program. The final follow-up session in week seven was completed either through a home visit or a phone call to evaluate the implementation of the strategies and provide final support.

Two program instructors who were occupational therapists led the group educational session in conjunction with an occupational therapy student. Individual programs and subsequent aspects were completed by one occupational therapist or an occupational therapy student.

An AUD30 gift card was given to all participants as a small acknowledgement of time spent.

### Pre- and post-program assessments

Demographic, background information, and pre-program assessments were collected at the beginning of the educational program. The post-program assessments and acceptability questionnaire were completed during or after the final home visit or phone call.

#### Demographic and background Information

Informal dementia carers recruited were asked for information regarding age, sex, country of birth, language, years of formal education, current occupation, years and weekly hours of care provided, their residential status and existing services accessed. Information was also collected regarding the care-recipients, either from the hospital record or from the informal dementia carers, including their age, sex, country of birth, preferred language, years of formal education, previous occupation, living situation, clinical frailty score^20^, clinical diagnosis, co-morbidities, and discharge destination. The clinical frailty score, collected within one month of data collection, was obtained from the hospital record.

#### Feasibility

The recruitment, retention and attendance of the program were recorded to indicate the program’s feasibility.

#### Acceptability

A questionnaire was used to capture the informal dementia carers’ acceptance of the educational program regarding the appropriateness, relevance, and practicality of the educational program. The questionnaire with four attributes, effectiveness, acceptability/logicality, suitability/appropriateness, and convenience was adopted from Francois, et al^21^. and was used in other studies to review the program acceptability^22,23^. The questionnaire was further categorized into location convenience, schedule and duration, content usefulness, understanding of the educational program, the follow-up phone calls and the take-home booklet. Each question was scored from one, ‘Completely Disagree’, to four, ‘Completely Agree’. The form was completed via phone, mail, or email.

#### Carer burden

Burden was measured using the Zarit Burden Interview (ZBI), a 22-question, paper-based self-reported questionnaire^24^ developed to be used with carers of people with dementia within the community^25^. This measure addresses specific factors linked to carer burden, such as level of assistance, perceived control over one’s life, impact on the individual carer, the social impact of caring, and the overall measure of perceived burden. The overall score interpretation ranges from zero to 88, from ‘little or no burden’ to ‘severe burden’. The form was completed either over the phone, by mail or by email. The ZBI had excellent test-retest reliability, and was strongly correlated with assessments such as the Burden Assessment Scale for dementia carers^26^.

#### Quality of life

Quality of life was measured by the Adult Carer Quality of Life Questionnaire (AC-QoL)^27^, a 40-question, paper-based self-reported instrument used to screen for quality of life. The tool uses sub-scales including support for caring, caring choice, caring stress, money matters, personal growth, sense of value, ability to care and carer satisfaction, highlighting the holistic impact of caring on an individual. The sub-scales are similarly scored from ‘low reported quality of life’ to ‘high reported quality of life’, with scores ranging from zero to 120. The AC-QoL was completed either over the phone, by mail or by email, per participant’s preference. The AC-QoL has demonstrated good validity with strong correlation with measures of burden and mental health, and good internal consistence for assessing dementia carers^28^.

#### Care-recipient’s performance in everyday tasks

The level of performance and assistance required in everyday tasks for care-recipients was measured through the Alzheimer’s Disease Cooperative Study- Activities of Daily Living Scale (ADCS-ADL)^29^. This is a 23-item paper-based questionnaire reported by the carer about the care-recipient’s performance covering a range of activities such as eating, dressing, cooking and hobbies and was used as an outcome measure to show changes in the performance of everyday tasks before and after the educational program. The scoring ranges from zero to 78, from profound severity to typical performance. The assessment was completed either over the phone, by mail or by email. The ADCS-ADL has been shown to have moderate to good test–retest reliability and strong support for known-groups validity^30^.

### Data analysis

Descriptive statistics were used to report the demographic and background information. The percentages of recruitment, retention and attendance were reported. The results of the acceptability questionnaire were reported by medians. The medians and interquartile range (Q1-Q3) of the ZBI, AC-QoL and ADCS-ADL, collected before and after the program, were reported. In view of the small sample size, the non-parametric Wilcoxon signed-rank tests were used to investigate the differences, if any, between pre- and post-program results. Significance levels were set at smaller than or equal to 0.05. All analyses were performed using the Statistical Package for Social Sciences (SPSS) version 25.0.

## Results

### Feasibility

From the 65 informal dementia carers approached, 16 participant dyads were recruited over a six-month period, with 14 of them recruited while the care-recipients were hospitalized. The recruitment rate was 25%. The retention rate was 100% and no participants withdrew from the study throughout the program. However, two informal dementia carers missed one follow-up phone call during the seven-week period because of work commitments and COVID-19 changes. The attendance rate for the educational session was 100%, and the follow-up phone calls were 97%. All informal dementia carers recruited spoke in English.

The demographic and background information of the program participants (informal dementia carers and care-recipients) are displayed in Tables 1 and 2.

**Table 1:** Demographic information of the participant dyads (*N* = 16).

**Demographic information**	**Care-recipient**	**Caregiver**
Age, years, mean (SD)	81.63 (8.67)	59.13 (12.24)
Sex, n (%)		
Male	4 (25)	3 (18.80)
Female	12 (75)	13 (81.30)
Country of birth, n (%)		
Australia	5 (31.30)	8 (50)
Singapore	1 (6.30)	1 (6.30)
Laos	1 (6.30)	1 (6.30)
Malta	1 (6.30)	-
Italy	2 (12.50)	-
Egypt	1 (6.30)	1 (6.30)
England	2 (12.50)	2 (12.50)
Chile	1 (6.30)	1 (6.30)
Vietnam	1 (6.30)	1 (6.30)
Iran	1 (6.30)	1 (6.30)
Language spoken at home, n (%)		
English	11 (68.80)	-
Assyrian	1 (6.30)	-
Laotian	1 (6.30)	-
Greek	1 (6.30)	-
Italian	1 (6.30)	-
Vietnamese	1 (6.30)	-
Formal education, n(%)^1^		
No schooling	1 (8.30)	-
Primary school level	3 (25)	1 (6.30)
Year 9 - 10	4 (33.30)	6 (37.50)
Year 11 - 12	3 (25)	2 (12.50)
Tertiary	1 (8.30)	7 (43.80)
Previous occupation for care-recipients, n(%)		
Trade	4 (25)	-
Retail	4 (25)	-
Mixed	2 (12.50)	-
Managerial	2 (12.50)	-
Home duties	4 (25)	-
Current employment for caregivers, n (%)		
Full-timer carer	-	7 (43.80)
Full-time employment	-	3 (18.80)
Casual/Part-time employment	-	3 (18.80)
Not employed	-	3 (18.80)

^1^ Valid percentage excluding the missing data (n=4)

**Table 2:** Background information of the participant dyads (N=16).

**Background Information**	**Care-recipient**	**Caregiver**
Accommodation type of care-recipients, n(%)^1^		
Living with spouse	5 (31.30)	-
Living with children or family	6 (37.50)	-
Living alone	2 (12.50)	-
Living with spouse and children	2 (12.50)	-
Living with a friend	1 (6.30)	-
Carer living with care-recipients, n(%)		
Yes	-	10 (62.50)
Years living with care-recipients, mean (SD)	-	31.22 (21.89)
No	-	6 (37.50)
Years caring, mean(SD)	-	4.29 (3.91)
Number of hours caring per week, mean(SD)	-	118.66 (63.89)
Receiving home care services, n (%)		
Yes	10 (62.50)	-
No	6 (37.50)	-
Type of Dementia, n(%)		
Early Onset	2 (12.50)	-
Alzheimer’s disease	3 (18.80)	-
Mixed	2 (12.50)	-
Vascular	1 (6.30)	-
Frontotemporal	1 (6.30)	-
Not specified	7 (43.80)	-
Number of additional medical conditions, n (%)		
0	2 (12.50)	-
1-5	4 (25)	-
6-10	5 (31.30)	-
11-19	5 (31.30)	-
Discharge destination, n (%)^2^		
Home	11 (78.60)	-
Home with child	2 (14.30)	-
Respite	1 (7.10)	-
Rowland universal dementia assessment scale (RUDAS) Score /30, mean (SD)^3^	17.91 (4.39)	-
Clinical frailty scale, mean(SD)	5.81 (1.56)	-

^1^ Carers in the dyads may or may not live with the care recipient

^2^Valid percentage excluding the care-recipients who were referred from outpatient clinic (n=2)^3^ Data excluding the missing data (n=5)

## Acceptability

**Fig. 1:**
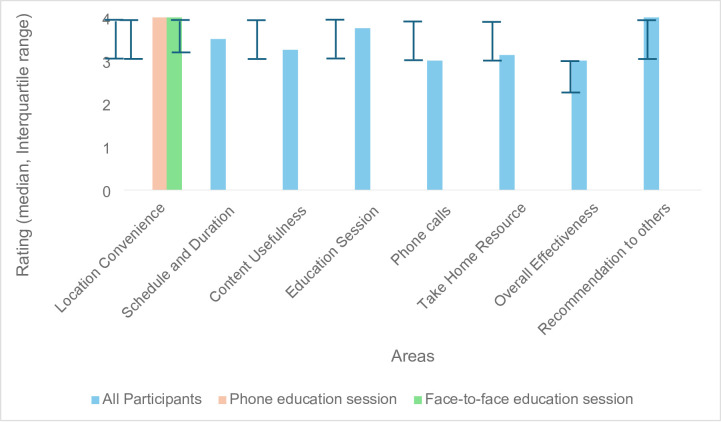
Results of the acceptability questionnaire. Ratings: 1 = Completely disagree, 2 = Disagree, 3 = Agree, 4 = Completely Agree.

Figure 1 illustrates the results of the acceptability questionnaire. All informal dementia carers rated the program as positive, with the median score of each area rated as ‘agree’ or ‘completely agree’ (Fig. 1). The convenience of the location and likelihood of recommending the program to other carers received the median score of 4 showing the highest level of satisfaction.

## Effectiveness

Table 3 shows the results of the assessments pre- and post-program. No statistically significant findings were ascertained in the ZBI before and after the educational program. For the AC-QoL, significant improvement was found in the Personal Growth subscale (*p* = 0.03) but not in the overall score. Apart from the Personal Growth subscale, improvement after the educational program were revealed in three items from other subscales, including improved feelings of being ‘worn out’ (*p* = 0.03), ability to save money (*p* = 0.03) and growth as a person (*p* = 0.02). There was also a decrease in quality of life in feelings of depression due to caring (*p* = 0.03), satisfaction with caregiving performance (*p* = 0.02) and satisfaction with the caregiving lifestyle (*p* = 0.02). For the results of the ADCS-ADL, improvements were noted in performance in paying attention to conversation (*p* = 0.03) and shopping (*p* = 0.02). Conversely, the ability to be left alone decreased (*p* = 0.04).

**Table 3:** Pre- and post-program assessment scores (*N* = 16).

**Assessments**	**Pre-program scores** Median (Interquartile range)	**Post-program scores** Median (Interquartile range)	*p*value
ZBI^b^	27.50 (17.25-45.25)	34 (22.50-51.50)	0.26
AC-QoL^a^	76.50 (55.25-95)	70 (57.50-95.75)	0.45
Support for caring	8.50 (4.50-10)	9 (6-11)	0.22
Caring choice	9.50 (4-11.75)	10 (5.25-12)	0.45
Caring stress	9 (5.25-11.75)	10 (5.75-11)	0.96
Money matters	7 (5.25-9.75)	7 (5-11)	0.72
Personal growth	10 (6.25-12.75)	10.50 (7-13.75)	0.03*
Sense of value	8.50 (6-14.75)	11 (6.25-13.75)	0.75
Ability to care	11 (9.25-14)	10.50 (8.25-12.75)	0.27
Carer satisfaction	12.50 (7.50-14)	10 (7-12)	0.14
ADCS-ADL^a^	26.50 (14.25-48)	17 (14-45.75)	0.88
1 Eating	3 (2-3)	2.50 (2-3)	1.00
2 Walking	2 (0-3)	2 (0-3)	0.85
3 Toileting	0.50 (0-3)	1 (0-2.75)	0.89
4 Bathing	1 (0-2)	0 (0-2)	0.16
5 Grooming	2 (0.25-2.75)	2 (1-2)	0.61
6 Dressing-			
a Picking out clothes	1 (0-3)	1.50 (0-2.75)	0.67
b Physically getting dressed	1.50 (0-3.75)	0 (0-4)	0.33
7 Telephone	1.50 (0-3)	1 (0.25-2.75)	0.76
8 Television	1 (0-2)	1 (0-2)	0.71
9 Conversation	0.50 (0-3)	2.50 (1.25-3)	0.03*
10 Dishes	0 (0-2.25)	0 (0-0)	0.28
11 Personal belongings	0.50 (0-2.75)	1 (0-1.75)	0.72
12 Drink	0.50 (0-3)	1 (0-3)	1.00
13 Cooking snacks	0 (0-2.75)	0 (0-0.75)	0.11
14 Litter	1.50 (0-3)	2 (0-3)	0.83
15 Travel	0 (0-2)	1 (0-2)	0.43
16 Shopping (including paying)	0 (0-0.75)	0.50 (0-3.50)	0.02*
17 Appointments	1 (0-2)	1 (0.25-2)	0.52
18 Alone	0 (0-2)	0 (0-0)	0.04*
19 Current events	0 (0-2)	0 (0-1)	0.60
20 Reading	0 (0-0)	0 (0-0)	1.00
21 Writing	0 (0-0.75)	0 (0-0.75)	1.00
22 Hobbies	1 (0-3)	0 (0-3)	0.19
23 Appliances	0 (0-3.75)	0 (0-1.50)	0.14

* indicates statistical significance *p* ≤ 0.05

^a^A higher score indicates better performance;^b^a lower score indicates better performance

## Discussion

Our findings support the use of an everyday-task-focused, strategy-based educational program for informal dementia carers. The program encompasses a comprehensive range of meaningful activities for older adults and employed a strategy-based approach grounded in the principles of Montessori for Dementia and Ageing^19,31,32^. The intervention appears to be both feasible and highly acceptable, as evidenced by positive feedback from participating informal dementia carers. Furthermore, the results provided preliminary evidence of the program’s effectiveness in enhancing carers’ quality of life and care-recipients’ participation in everyday tasks, although no significant reduction in carers’ perceived burden was observed. These findings align with those of Cooper, et al^33^., who reported that educational programs incorporating tailored strategies and meaningful activities for carers and care-recipients contribute to greater carer satisfaction.

Regarding feasibility, the study achieved a comparatively low recruitment rate of 25%. However, it is important to recognize that recruitment rates in research contexts may not accurately reflect the feasibility of implementing such educational program in real-world settings, as potential participants may be deterred by the research component rather than the intervention itself^34^. Several factors may have influenced recruitment, including possible reluctance to disclose the dementia diagnosis^35^, a low rate of dementia diagnosis due to its perceived negative impact, and the availability of alternative educational programs for informal dementia carers, and the need for clearer communication about the unique benefits of our program. Joshi, et al^36^.suggest strategies such as building trusting relationships with potential participants providing detailed information about the individualized benefits of the program to enhance recruitment. Despite the modest recruitment rate, our study demonstrated an excellent attendance (100%) and retention rate (100% for the educational session and 97% for the follow-up phone calls), when compare favorably to previous studies (e.g., Teles, et al^37^. reported attendance and retention rates of 78% and 74% respectively). These results suggested that, while the educational program is attractive and engaging for those who enroll, future studies should focus on optimizing recruitment strategies to reach a broader population of informal dementia carers.

The high levels of acceptability reported by participants may be partially explained by the isolating nature of caregiving^38^ and lack of social support often experienced by informal carers^39^. The presence of a supportive healthcare professional who listens to carers’ challenges and offer practical strategies can significantly enhance the caregiving experience. Zarit, et al^40^. found that program intensity and stress levels of carers involved in their program did not necessarily correlate with the acceptability scores, a finding echoed in our study, where no direct relationship was observed between burden, quality of life outcomes, and acceptability. This suggests that the perceived value of the program may be more closely related to the general support and increased knowledge provided, rather than direct changes in burden or quality of life. Additionally, external factors such as the ongoing impact of the COVID-19 pandemic may have influenced participants’ perceptions of burden and quality of life. In regard to the program delivery, while face-to-face sessions offer opportunities for interpersonal interactive and sharing of experiences, phone-based sessions may be more accessible for carers with significant caregiving or work commitments. The high likelihood of participants recommending the program to others further underscores its perceived usefulness and relevance to carers’ needs.

The primary objective of this study was to assess feasibility and acceptability of an everyday-task-focused, strategy-based educational program for informal dementia carers, with a secondary aim to explore its effectiveness. While effectiveness was also explored, it is important to acknowledge that, as a pilot study with small sample size, the statistical power to detect significant changes was limited^41^. This limitation is reflected in our findings, which did not demonstrate a significant reduction in carer burden. In contrast, larger studies have reported positive effects of educational programs on carer burden. For instance, a systematic review and meta-analysis encompassing 395 participants across five randomized controlled trials found a moderate effect of education on carer burden (SMD = 0.52; 95% confidence interval = 0.79 to 0.26; I^2^ = 40%)^42^. These studies benefit from greater statistical power and more diverse samples, which may account for their ability to detect significant changes.

It is also important to consider the measurement tools used. The ZBI, employed in this study, captures a broad spectrum of burden, including emotional, social, and financial aspects, rather than focusing exclusively on the impact of everyday caregiving tasks. While these aspects may be intertwined, interventions that primarily target practical caregiving strategies may not fully address the broader psychosocial dimensions of burden. Additionally, factors such as the stage of dementia, the presence of behavioral and psychological symptoms, and the availability of external support services can all influence carers’ perceived burden, potentially diluting the observable impact of the intervention.

Regarding informal dementia carers’ quality of life, our study identified a notable improvement in the domain of personal growth. This finding is significant, as it suggests that the program may foster adaptive coping mechanisms among carers. Biggs, et al^16^. describe “proactive coping” as the process by which individuals actively seek resources and opportunities to enhance their personal growth and resilience. The educational program provided carers with the opportunities to reflect on their caregiving experiences, acquire new skills and implement strategies, thereby promoting confidence and a sense of empowerment. Improvements in feelings of being ‘worn out’ further suggest that the strategies taught may have alleviated some of the physical and emotional demands associated with caregiving. However, lower levels of satisfaction with performance and lifestyle may reflect ongoing challenges, such as limited access to respite services and increased strain due to the prevalence of behavioral and psychological symptoms in care-recipients^43^.

Despite these positive outcomes in specific domains, our study did not observe an improvement the overall quality of life score, diverging from findings in previous research as as Thomas, et al^7^.. This discrepancy is likely attributable to the small sample size and multifactorial nature of quality of life, which is influenced by a range of external factors – including financial stressors, social isolation, and the broader impact of the COVID-19 pandemic – that may not be directly addressed by the intervention. Future studies with larger, more diverse samples and longer follow-up periods are needed to more accurately assess the program’s impact on overall quality of life.

Importantly, carers reported improvements in care-recipients’ attention to conversation and engagement in shopping, showing that the educational program may have positively influenced participation in meaningful activities. Ripich, et al^44^. suggest the potential for people with dementia to maintain conversational skills when supported by appropriate communication strategies. The inclusion of such strategies in our educational program may have been facilitated greater engagement and interaction, contributing to improved participation in everyday tasks. Nevertheless, it is important to recognize the progressive nature of dementia, and therefore, performance in everyday tasks inevitably deteriorates over time^1^. This progression may limit the sustainability of improvements in participations, particularly in community-based activities such as attending day programs or social appointments.

The interpretation of our findings must also consider several confounding factors. Due to the unique situations of each informal dementia carer and the individual nature of any one person’s experience of dementia, there are a range of confounding factors influencing the findings. The various stages of dementia that were present throughout the care-recipients could have impacted the effectiveness of the program. For our care-recipients, the average frailty was ‘mildly frail’, with a wide range of frailty status for the people living with dementia. Those in the earlier stages of dementia may have not seen immediate impacts of the strategies, while later stages may have already implemented their own strategies or may be unable to implement strategies due to limited abilities. There was a small sample size, as is appropriate for a pilot intervention, and the informal dementia carers were from a similar geographic location, which may limit the generalizability of these findings to other population groups. The absence of blinding and a control group also increases the likelihood of bias influencing the findings. Nonetheless, we found that this program was feasible, acceptable with preliminary effectiveness shown.

### Study limitations

The single-group, pre- and post-test study design, small sample size, and use of convenience sampling limit the generalizability of the findings to the broader population of individuals caring for someone with dementia. Consequently, conclusions regarding the program’s effectiveness should be interpreted with caution and require replication and further investigation in larger, more representative samples of dementia carers and care-recipients. Nevertheless, these methodological limitations do not preclude the use of the data for feasibility assessment of the everyday-task-focused, strategy-based educational program. Additionally, reliance on self-reported measures of carer burden, quality of life, and depression may have influenced participant responses, as these instruments can be lengthy and may be perceived as formal, intrusive, or irrelevant by some carers^9^. Such factors could have affected the accuracy and reliability of the reported outcomes, thereby impacting the study’s findings.

### Clinical and research implications

Our findings indicate that an everyday-task-focused, strategy-based educational program is both feasible and acceptable, with promising preliminary signals of efficacy in a small group of 16 carer-care-recipient dyads. This study contributes to the growing repertoire of practical educational interventions available to clinicians and researchers, highlighting the value of combining in-person and phone sessions, providing accompanying resources, and utilizing a strategy-based approach with concrete strategy examples. This program provides practical strategies for addressing difficulties with everyday tasks in an easily accessible and flexible format. Continuing research using a large-scale randomized controlled trial, with blinded participants and assessors, across different locations is essential to define the effects of this program. Additional time-points to assess long-term outcomes will provide a better understanding of the impacts of the educational program. In the future, the educational program may be developed into a train-the-trainer model to increase accessibility for informal dementia carers.

## Conclusion

This study provides initial evidence supporting the feasibility, acceptability and potential benefits of an everyday-task-focused, strategy-based educational program in improving the quality of life for carers and participation in everyday tasks for people with dementia. This study demonstrated the importance of a comprehensive and individualized support model, utilizing a collaborative approach and practical training, which may help carers manage daily challenges, potentially reduce burden and improve psychological well-being^45^. While the results are preliminary, they offer useful insights for future study aimed at confirming the effectiveness of this educational program and refining its delivery to maximize impact for carers and care-recipients.

## Supplementary Information

Below is the link to the electronic supplementary material.

Supplementary Material 1

## Data Availability

Data is available on request from the corresponding author (KL).
