# Supplementary material for: An everyday-task-focused, strategy-based educational program for informal dementia carers: a feasibility and pilot study
Source: Sci Rep. 2026 Jan 20;16:5863. doi: 10.1038/s41598-026-36887-3 (PMC12894958; doi:10.1038/s41598-026-36887-3)
Supplement: Supplementary file 1 — Supplementary Material 1 [file 41598_2026_36887_MOESM1_ESM.docx]

**Appendix A**

**Educational Program Content Validation**

**Methods**

***Participants*.** Validity of the program’s content was assessed through an expert panel review. Fourteen occupational therapists were recruited through purposive and convenience sampling from the local health district occupational therapy aged care network group. They were all practicing occupational therapists, aged 18 years and above, had 3 or more years of experience in occupational therapy and 1 or more years of experience in geriatrics (Table A).

**Table A** Expert Panel Demographic Information

| **Demographic** | **Number of participants (%)** |
| --- | --- |
| *Gender* |  |
| Female | 13 (92.9) |
| Male | 1 (7.1) |
| *Age* |  |
| 18-29 | 4 (28.6) |
| 30-39 | 9 (64.3) |
| 40+ | 1 (7.1) |
| *Qualification Level* |  |
| Bachelor | 9 (64.3) |
| Masters | 5 (35.7) |
| *Occupational Therapy Years of Experience* |  |
| 3-5 | 6 (42.9) |
| 6-10 | 2 (14.3) |
| 11-15 | 3 (21.4) |
| 16+ | 3 (21.4) |
| *Geriatric Years of Experience* |  |
| 1-5 | 7 (50.0) |
| 6-10 | 5 (35.7) |
| 11+ | 2 (14.3) |
| *Practice Setting* |  |
| Inpatient Acute | 8 (57.1) |
| Inpatient Rehabilitation | 1 (7.1) |
| Community | 4 (28.6) |
| Research | 1 (7.1) |

*Note*. Percentages are rounded to 1 decimal place.

***Data collection*.** Expert panel members were presented with a detailed outline of the educational program, including the components of the program, topics covered and strategy-based approach. A copy of the written resource was provided for reference throughout the review.

Expert panel members were requested to complete a questionnaire to review the program. Each question was targeted to one of four attributes as adopted from Francois, Lanier, Marich, Wallendorf, and Van Dillen (2018). The attributes included (1) perceived effectiveness – the program is useful for carers; (2) relevancy – the program is appropriate to carers; (3) convenience – the program’s format is convenient for carer participation; and (4) clarity – the program is logical and easy to understand. The questions were also targeted towards one of four features of the program (a) face-to-face sessions, (b) phone sessions, (c) accompanying resource, and (d) overall program. These components were examined through a 5-point Likert scale where ‘1’ represented ‘poor’ and ‘5’ represented ‘excellent’. Panel members were encouraged to provide additional comments or suggestions, particularly if they provided a ‘1’ or ‘2’ rating [The questionnaire was adopted and used as the Acceptability Questionnaire in the pilot study].

***Data analysis.*** The content validity index (CVI) was adopted to analyse the numerical data from the expert panel. The CVI identifies aspects that need to be revised and improved [1]. Researchers followed the process as specified by Polit, et al. [2]. For each question, an individual content validity index (I-CVI) was calculated by collating all positive ratings, ‘3’ or above, and dividing by the total number of panel members. All I-CVIs were averaged for each attribute (perceived effectiveness, relevancy, convenience and clarity) and each feature (face-to-face sessions, phone sessions, accompanying resources and overall program) to gain an attribute content validity index (A-CVI) and feature content validity index (F-CVI) respectively. Receiving a score higher than 0.78 indicates an acceptable content validation score (Lynn, 1986; Polit et al., 2007). Written and verbal comments provided by the members were collated and categorised. Both numerical data and comments were analysed to revise the program.

**Results**

**Numerical data.** The I-CVIs for all questions are displayed in Table B. All questions were above the acceptable validation score of 0.78, receiving either 0.93 or 1.00. All panel members agreed on the attributes; perceived effectiveness, relevancy, convenience and clarity (A-CVI = 0.98, 0.99, 0.97 and 0.98 respectively). They also agreed for all features assessed; face-to-face sessions, phone sessions, accompanying resources and overall program (F-CVI = 0.98, 1.00, 0.99 and 0.95 respectively).

**Table B** Content Validation Questions and Index Scores

| **Question** | | **Attribute Assessed** | **Feature Assessed** | **I-CVI** |
| --- | --- | --- | --- | --- |
| 1 | Is the program structure with 4 face-to-face sessions and 2 phone sessions appropriate to achieve the aim of the educational program? | Perceived Effectiveness | Overall Program | 1.00 |
| 2 | Is the duration of the program including 4 face-to-face sessions (1 hour each) and 2 phone sessions (20 minutes each) appropriate? | Relevancy | Overall Program | 0.93 |
| 3 | Is the program structure and location (4 face-to-face sessions at a hospital and 2 phone sessions at home) feasible and convenient for carer attendance / availability? | Convenience | Overall Program | 0.93 |
| 4 | In the face-to face session, are the topics presented helpful? | Perceived Effectiveness | Face-to-face Sessions | 1.00 |
| 5 | Are the topics presented in the program relevant to the carer? | Relevancy | Face-to-face Sessions | 1.00 |
| 6 | In the face-to-face session, are the activities provided appropriate for carers? | Relevancy | Face-to-face Sessions | 1.00 |
| 7 | In the face-to-face session, are the instructions provided easy to follow? | Clarity | Face-to-face Sessions | 0.93 |
| 8 | In the phone sessions, are the questions appropriate for carers? | Relevancy | Phone Sessions | 1.00 |
| 9 | In the phone sessions, are the instructions provided easy to follow? | Clarity | Phone Sessions | 1.00 |
| 10 | Does the program content and format seem effective in improving ease of care and reducing carer burden? | Perceived Effectiveness | Overall Program | 0.93 |
| 11 | Is the resource content acceptable and logical for carers? | Clarity | Accompanying Resources | 0.93 |
| 12 | Are the written instructions clear and easy to understand? | Clarity | Accompanying Resources | 1.00 |
| 13 | Does the layout of the Take Home Resource facilitate easy location of information? | Convenience | Accompanying Resources | 1.00 |
| 14 | Are the strategies clear? | Clarity | Accompanying Resources | 1.00 |
| 15 | Are the strategies practical and useful? | Perceived Effectiveness | Accompanying Resources | 1.00 |
| 16 | Are the activities included appropriate for carers? | Relevancy | Accompanying Resources | 1.00 |
| 17 | Are the activities included easy to follow and clearly stated? | Clarity | Accompanying Resources | 1.00 |

**Comments.** Panel members commented that the educational program’s content was very appropriate and necessary for the target population. The phone sessions in particular were deemed convenient and relevant, with a good open structure to facilitate a two-way conversation. The panel members responded positively to the use of a strategy-based approach and the list of strategy examples presented in the resource. Some panel members expressed concern at the complexity of various topics such as the ‘strategy generation framework’, but thought the inclusion of examples and activities enhanced understanding.

Panel members expressed concern at the convenience of the program, particularly in regards to the four face-to-face sessions. It was considered by many members that although literature suggested a high amount of interactions [3], this would not be a feasible option for carers. This is due to their daily care requirements, limited time and difficulty receiving respite. The phone sessions were rated as very convenient for carers and there were recommendations to extend this feature of the program. Suggestions also included scheduling face-to-face sessions during a care recipient’s inpatient hospital stay or one-on-one sessions at a carer’s home.

Participants stated that the written and verbal instructions would be understandable for native English speakers. However, participants expressed concern for lack of understanding with populations who are culturally and linguistically diverse, have low literacy or cognitive impairments. Language specific groups or audio-visual resources were suggested to enhance verbal communication. Suggestions for written education included adding a glossary or creating basic English or translated versions of the resource.

Another common theme in participant’s comments was that the 50-page resource may be overwhelming for carers. Splitting the resource into multiple smaller resources and including pictures, flow charts and tables was suggested.

Participants identified the need to follow up with more complex strategies such as home modifications or adaptive equipment. Changing the phone sessions to take place after face-to-face sessions, making referrals to community occupational therapists and including links to further resources was suggested to minimise this limitation.

**Revisions to program** (Table C). Using results from the expert panel review, revisions were made to the educational program before commencement of the pilot study. Notably, the format was changed to one educational session with a break in between taking place on the same day, three follow up phone sessions, and a final session conducted either by a home visit, online, or by phone. The accompanying resource was split into two parts with pictures to illustrate concepts. There were opportunities to include a translator for participants of culturally and linguistically diverse backgrounds.

**Table C** Revision of the Program Following Expert Panel Review

| **Related Topic** | **Before Expert Panel Review** | **After Expert Panel Review** |
| --- | --- | --- |
| Convenience of the program | Four face-to-face sessions | One face-to-face session (with a break), three follow up phone sessions, and a home visit or phone call |
| Informal Dementia Carer Strategies Package | 50-page resource | Resource divided into sections, with pictures to illustrate concepts |
| Strategy generation framework | Framework only | Framework supplemented with examples |
| Language |  | Added glossary of terms in different languages |
|  |  | Translator provided for participants from culturally and linguistically diverse backgrounds, if needed |

**References**

1. Delgado-Rico E, Carretero-Dios H, Ruch W. Content validity evidences in test development: An applied perspective. International Journal of Clinical and Health Psychology 2012;**12**(3):449-60.

2. Polit DF, Beck CT, Owen SV. Is the CVI an acceptable indicator of content validity? Appraisal and recommendations. Research in Nursing and Health 2007;**30**(4):459-67 doi: 10.1002/nur.20199.

3. Laver K, Cumming R, Dyer S, et al. Evidence-based occupational therapy for people with dementia and their families: What clinical practice guidelines tell us and implications for practice. Australian Occupational Therapy Journal 2017;**64**(1):3-10 doi: 10.1111/1440-1630.12309 [published Online First: 20161003].

**Informal Dementia Carer Education Program**

Informal dementia carers will participate in a seven-week educational program involving one educational session, three follow-up phone calls, and a home visit or phone call.

The two-hour face-to-face group educational session (with a break in between) will occur with a group of 2 to 4 informal dementia carers. The group format will facilitate sharing of caring concerns among carers. During the session, the strategy framework, with the list of strategies, will be explained (please refer to p. 2 of this document). They include strategies from four categories; adapt, engage, orientate or using senses. The strategies are also grouped by the types of everyday tasks, with additional general strategies and strategies focussing on aggression, agitation, and hallucinations.

Examples of applying the strategies in assisting with everyday tasks will be worked through with the informal dementia carers. This process is then applied to informal dementia carers’ individual concerns, working collaboratively with healthcare professionals or program instructors to solve problems with assistance for everyday tasks. During the session, the *Informal* *Dementia Carer Strategies Package* on the strategy framework, the list of strategies and examples on how to use them are given to the informal dementia carers to refer to. A schedule of the program and a journal to record their experiences to be used in the subsequent weeks are provided.

Following the group educational session, participating carers will receive three 20-minute follow-up phone calls in weeks two, four and six of the program. The phone calls aim to provide additional support and gauge how they use the strategies. Lastly, the program will be completed with a final home visit, or phone call to evaluate the implementation of the strategies and provide final support.

**Informal Dementia Carer Strategies Package Outline**

***Strategy Framework***


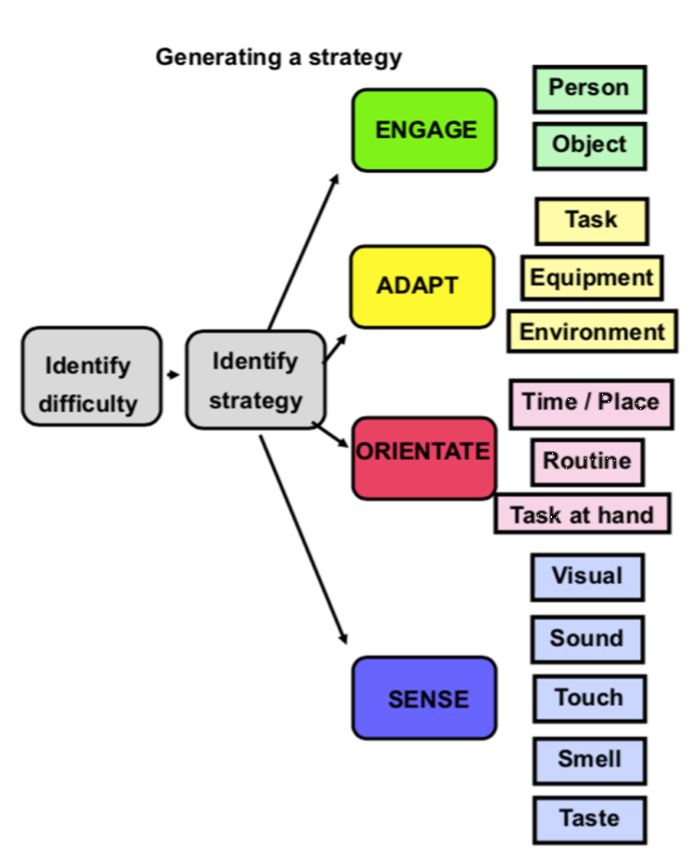


**ENGAGE**: is finding strategies that will increase the care recipient’s interest in different activities or their entire day.

**ADAPT**: is finding strategies that alter either the **task**, **equipment** or **environment**.

**ORIENTATE**: is using strategies that focus on orientating your care recipient.

**SENSE:** is using our five senses to create strategies we can use while caregiving.
